# The effects of combined high-frequency repetitive transcranial magnetic stimulation and cervical nerve root magnetic stimulation on upper extremity motor recovery following stroke

**DOI:** 10.3389/fnins.2023.1100464

**Published:** 2023-02-07

**Authors:** Xiaofang Wu, Rui Wang, Qunqiang Wu, Chunhua Liao, Jianshe Zhang, Huiduo Jiao, Baolin Chen, Shuyan Wang, Rui Liu

**Affiliations:** ^1^Department of Rehabilitation, Tangdu Hospital, Air Force Medical University, Xi’an, China; ^2^Graduate School of Xi’an Medical College, Xi’an, China; ^3^Medical Department of Tangdu Hospital, Air Force Medical University, Xi’an, China

**Keywords:** repetitive transcranial magnetic stimulation, stroke, upper limb motor function, rehabilitation, cervical nerve root

## Abstract

**Introduction:**

Upper limb motor impairments after stroke cause patients partial or total loss of the capability of performing daily living, working, and social activities, which significantly affects the quality of life (QoL) of patients and brings a heavy burden to their families and society. As a non-invasive neuromodulation technique, transcranial magnetic stimulation (TMS) can act not only on the cerebral cortex, but also on peripheral nerves, nerve roots, and muscle tissues. Previous studies have shown that magnetic stimulation on the cerebral cortex and peripheral tissues has a positive effect on the recovery of upper limb motor function after stroke, however, few studies have reported the combination of the two.

**Objective:**

This study was to investigate whether high frequency repetitive transcranial magnetic stimulation (HF-rTMS) combined with cervical nerve root magnetic stimulation more effectively ameliorates upper limb motor function in stroke patients. We hypothesized that the combination of the two can achieve a synergistic effect and further promotes functional recovery.

**Methods:**

Sixty patients with stroke were randomly divided into four groups and received real or sham rTMS stimulation and cervical nerve root magnetic stimulation consecutively before other therapies, once daily over five fractions per week for a total of 15 times. We evaluated the upper limb motor function and activities of daily living of the patients at the time of pre-treatment, post-treatment, and 3-month follow up.

**Results:**

All patients completed study procedures without any adverse effects. The upper limb motor function and activities of daily living improved in patients of each group were improved after treatment (post 1) and 3 months after treatment (post 2). Combination treatment was significantly better than single treatments alone or sham.

**Conclusion:**

Both rTMS and cervical nerve root magnetic stimulation effectively promoted upper limb motor recovery in patients with stroke. The protocol combining the two is more beneficial for motor improvement and patients can easily tolerate it.

**Clinical trial registration:**

https://www.chictr.org.cn/, identifier ChiCTR2100048558.

## 1. Introduction

Stroke is a common disease that seriously threatens human health. In recent years, the incidence of stroke has been increasing and gradually showing a younger trend. According to statistics, approximately 85% of stroke survivors have upper extremity dysfunction in the early stage (Naghavi et al., 2017). Although receiving conventional rehabilitation, such as physical therapy (PT), occupational therapy (OT), acupuncture and massage, a considerable number of patients still suffer from varying degrees of upper extremity motor dysfunction (Winstein et al., 2016). In the early stage after stroke, the main symptoms are limb paralysis and sensory disturbance, and in the later stage, limb spasm, pain, decreased coordination and flexibility may occur. The upper limb responsible for the complex, dexterous and coordinated motion, and usually it has slower recovery rate compared to the lower limb (Micera et al., 2020). Upper limb dysfunction caused by stroke make patients complete or partial loss the ability of daily living, which impose a great burden on the family and society, and it has always been the focus and difficulty of rehabilitation. Transcranial magnetic stimulation (TMS) is a non-invasive neuromodulation technique that induces currents in local areas of the cerebral cortex through brief, high-intensity magnetic fields to alter neuronal activity in the cerebral cortex and to promote neuroplasticity (Klomjai et al., 2015). Repetitive TMS (rTMS) refers to multiple TMS pulses given consecutively. Being painless, safe, effective, easy to operate, and simple, TMS has been widely applied in clinics nowadays. TMS can act not only on the cerebral cortex but also on peripheral nerves, nerve roots, and muscle tissue, which is also called peripheral magnetic stimulation (PMS) or functional magnetic stimulation (FMS) (Rossini et al., 2015). Li et al. (2016) found that low-frequency repetitive transcranial magnetic stimulation (LF-rTMS) and high-frequency repetitive transcranial magnetic stimulation (HF-rTMS) can significantly decrease the cortical latency of motor-evoked potentials (MEPs) and central motor conduction time, and improve upper-limb motor function in patients with cerebral infarction. Yang et al. (2021) reported that HF-rTMS (5 Hz) applied over the ipsilesional M1 for 10 days enhances hand functional recovery in subacute adult stroke patient. A randomized controlled trial (Jiang et al., 2022) showed that rPMS of upper limb extensor can improve arm function and muscle strength of stroke patients, and achieve grip strength and elbow flexion and extension. Although rTMS and PMS are both beneficial to improve upper limb motor function and daily living activities of patients with stroke, few studies have reported the efficacy of the combination of the two (Kumru et al., 2017; Gao et al., 2020). Neuromodulation of stroke should focus not only on the cortex but also on the nerve roots, and peripheral nerves. The stimulation of the cortex can top-down modulate neural plasticity, peripheral stimulation can bottom-up provide motor and sensory input, and the combination of the two forms a circuit to more effectively promote the recovery of neurological function after stroke. Based on this, the present study aimed to investigate the effects of the protocol that repeated HF-rTMS combined with cervical nerve root magnetic stimulation and provide a basis for clinical treatment.

## 2. Materials and methods

### 2.1. Participant

Sixty patients with upper limb hemiplegia after stroke who met the inclusion criteria and were hospitalized in the Rehabilitation Department of Tangdu Hospital from June 2021 to May 2022 were selected as the study subjects.

We included patients with (1) the first onset, meeting the diagnostic criteria for stroke (Zhang et al., 2020), (2) a unilateral lesion, confirmed by CT or MRI as a hemorrhagic or ischemic lesion in basal ganglia region; (3) the course of the disease is 2 weeks to 6 months, male or female, 30–80 years old; (4) conscious and the vital signs were stable; (5) has upper limb dysfunction and Brunnstrom stage I∼III of the affected upper limb; (6) willing to recover actively and able to cooperate with treatment instructions; (7) no mental abnormality; (8) the patient and/or the patient’s family members are authorized to understand and sign the informed consent.

The exclusion criteria were as follows: (1) patients with severe cognitive impairment or mental illness who cannot cooperate with treatment and evaluation; (2) intracranial metal foreign body; (3) history of epilepsy or family history of epilepsy; (4) pacemakers, stents, and cochlear implants; (5) those who cannot tolerate treatment; (6) have serious liver or kidney disease; (7) unable to cooperate with follow-up.

The demographic and clinical characteristics of the patients are summarized in Table 1.

**TABLE 1:** Comparison of basic data of patients.

Characteristics	Group A	Group B	Group C	Group D	F/x^2^/H	*p*
Cases (*n*)	15	15	15	15		
Age ($\bar{\text{x}}$ ± s, years)	54.60 ± 11.16	57.00 ± 10.76	54.87 ± 11.60	55.33 ± 10.30	0.15	0.93
Gender (Male/female, *n*)	10/5	12/3	11/4	15/0	6.49	0.10
BMI ($\bar{\text{x}}$ ± s, kg/m^2^)	23.76 ± 4.59	24.55 ± 4.63	25.55 ± 2.75	25.40 ± 3.02	0.69	0.56
Type of stroke (hemorrhagic/ischemic, *n*)	7/8	10/5	9/6	7/8	1.82	0.61
Course of disease (day)	38 (28,50)	34 (20,46)	26 (21,59)	32 (21,72)	0.46	0.79
Side of lesion (left/right, *n*)	9/6	12/3	9/6	5/10	6.78	0.08

BMI, body mass index; FMA-UE, Fugl-Meyer Assessment Upper Extremity Scale; WMFT, Wolf Motor Function Test; MBI, Modified Barthel Index.

### 2.2. Experimental design

**FIGURE 1:**
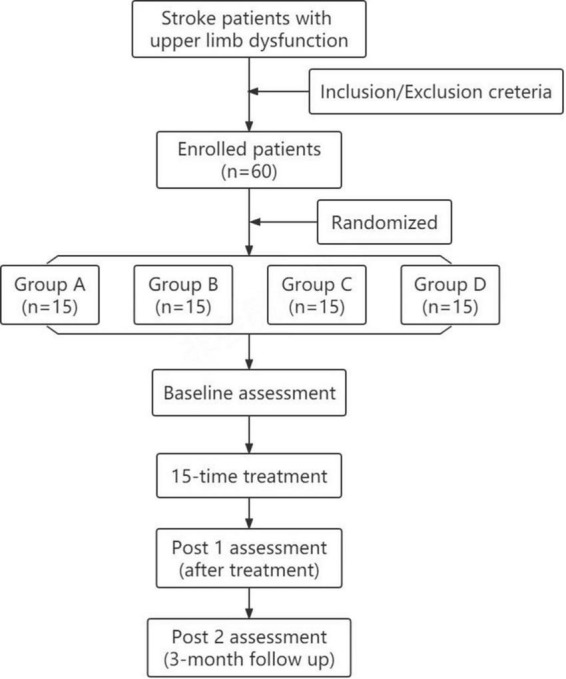
Flowchart showing recruitment, group allocation, allocation treatment, follow-up and analysis.

**FIGURE 2:**
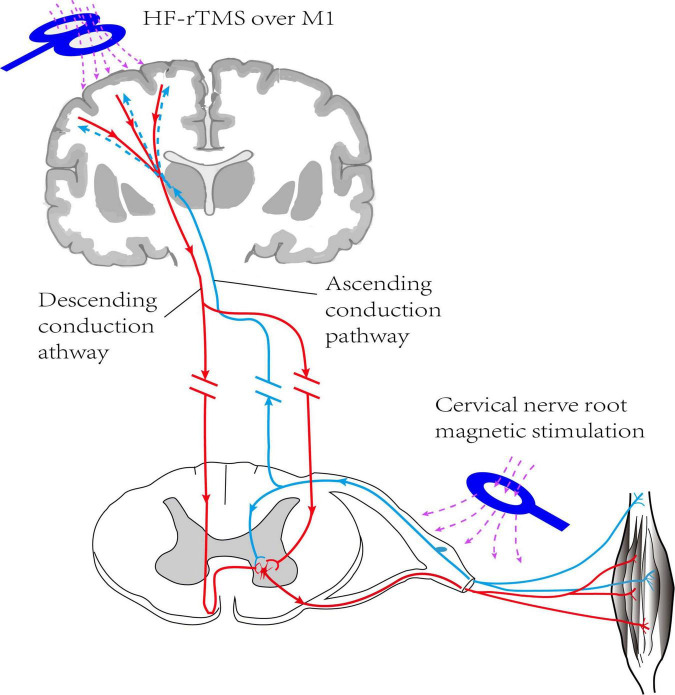
Schematic diagram of HF rTMS over ipsilateral M1 combined with magnetic stimulation of cervical nerve root on hemiplegic side (non-simultaneous). HF-TMS, high frequency repetitive transcranial magnetic stimulation; M1, primary motor cortex.

The study was a randomized, double-blind, sham-controlled trial, following the principle of randomization, control, and repeatability. As shown in Figure 1, the participants were randomly divided into four groups with 15 patients in each group. Group A, rTMS combined with cervical nerve root magnetic stimulation; Group B, only rTMS; Group C, only cervical nerve root stimulation; Group D, sham stimulation. Treatment was delivered once daily over five fractions per week for a total of 3 weeks. All patients underwent routine medical treatment and rehabilitation therapy during the implementation of treatment protocol. Motor function and daily living ability were evaluated at pre-treatment (baseline), post-treatment (post 1), and 3 months follow-up (post 2). The evaluation was performed by specially trained physicians who were unaware of the grouping and treatments. The person who performed the data analysis was also blinded. Figure 2 shows the schematic diagram of central and peripheral stimulation.

### 2.3. rTMS protocol

The rTMS protocols used in this study comply with the safety guidelines for rTMS applications (Lefaucheur et al., 2014; Rossi et al., 2021). In this study, all interventions were performed using a transcranial magnetic stimulator (CCY-I, YIRUIDE Medical Equipment Company, Wuhan, China). Brain stimulation was applied to the ipsilateral M1 using a 95 mm focal figure-of-eight coil and magnetic stimulation of the cervical nerve root were performed with a 125 mm circular coil. All participants receiving repetitive transcranial magnetic therapy used magnetic navigation software to exactly localize the optimal stimulation sites for rTMS.

Participants first underwent resting motor threshold (RMT) measurement to determine the intensity of treatment throughout the trial. RMT was defined as the minimum stimulation intensity needed to cause a MEP in the first dorsal interosseous muscle at least five of ten consecutive stimulations.

For group A, patients received rTMS at first and then went on to receive cervical nerve root magnetic stimulation. Repetitive TMS was delivered over the ipsilesional scalp site corresponding to the upper limb area of the primary motor cortex (M1), with the coil tangent to the hotspot. A total of 1,000 pulses of 10 Hz rTMS were applied, with the intensity at 80% of RMT (Kim et al., 2006) for 1 s followed by an inter-stimulus interval of 5 s. The target site of cervical nerve root magnetic stimulation is the cervical thoracic segment (C5-T1) of the hemiplegic side. Subjects received cervical nerve root stimulus protocol for 10 Hz consisting of 100 sequences of 10 pulses each, with 5 s between each sequence, for a total of 1,000 pulses, with a stimulation intensity to be the lowest stimulation intensity that can trigger muscle contraction (Matsumoto et al., 2013).

For group B, 10 Hz rTMS was performed over the ipsilateral M1 at 80% of RMT for a total of 1,000 pulses. Sham stimulation over the cervical nerve root was performed with the coil held at an angle of 90° to the hemiplegic side to reproduce the noise associated with the 10 Hz stimulus.

For group C, sham stimulation at ipsilateral M1 and real stimulation were performed over the cervical nerve root. The protocol of cervical nerve root stimulation is the same as that of group A. Sham stimulation was applied over ipsilateral M1 with the coil placed perpendicularly to the head.

For group D, the participants received sham stimulation at the same sites in the same order as group A. The coil was held at an angle of 90° to the hotspot so that patients could hear the sound but no actual stimulation effect.

### 2.4. Rehabilitation program

During the implementation of the protocol, all patients received conventional rehabilitation therapy composed of a 30-min of PT and a 30-min of OT, twice per day, five times per week for 3 weeks. PT includes a range of motion (ROM) training, anti-spasm training, muscle strength training, posture control training, balance and coordination training, etc. OT is mainly task-oriented functional training.

### 2.5. Observation indicators

The Fugl-Meyer Assessment of the Upper Extremity (FMA-UE) Scale (Hernandez et al., 2019) and the Wolf Motor Function Test (WMFT) (Bornheim et al., 2020) were used to measure the improvement of the upper limb and hand function in patients. FMA-UE included 10 major events, and 33 minor events, such as voluntary movement, reflex activity, grasp, and coordination, with a total score of 66 points. The higher the score, the better the Upper limb motor function. WMFT consists of 15 events, from 1 to 6 are simple joint movements, and from 7 to 15 are complex functional movements. The lowest score for each task is 0, and the highest score is 5, with a total score of 75. Higher scores represent a better function.

Modified Barthel Index (MBI) (Ohura et al., 2017) was used to measure the patient’s activities of daily living (ADL), which include 10 items, such as eating, grooming, dressing, transfer, et. The total score was 100. The scoring standard is as follows: ≤20 were totally dependent in life; 21–40 were classified as severe dysfunction and obvious life dependence; 41–59 were classified as moderate dysfunction and need some help with activities of daily life; ≥60 were classified as mild dysfunction and able to care for themselves in basic ADL; 100 were completely independent.

We use Brunnstrom motor recovery stage of the upper extremity and hand (Ikbali Afsar et al., 2018) to evaluate the motor development of hemiplegic patients. Brunnstrom staging criteria are as follows: stage I, muscle retardation, no reflex; stage II, mild spastic, with minimal voluntary movement; stage III, spasticity aggravates and can cause cooperative movement at will; stage IV, spasticity begins to decline, and can make a separated movement; stage V, establishment autonomous movement; stage VI, movement is close to normal.

### 2.6. Statistical analysis

Data conforming to the normal distribution are presented as mean ± standard deviation. Repeated measures analyses of variance (ANOVA) were used for multiple group comparisons. When the ANOVA revealed an interaction between time and intervention method, *post hoc* multiple pairwise comparisons were made with the Bonferroni correction. M(QR) was used for measurement data that did not conform to a normal distribution, the Kruskal–wallis rank sum test was used for inter-group comparison, and the Wilcoxon sign rank sum test was used for intra-group comparison; Classification data and grade data were expressed by constituent ratio or rate, and rank sum test was used for inter-group comparison. A *p*-value less than 0.05 was considered to be statistically significant.

## 3. Results

### 3.1. The outcome of upper extremity motor function and daily living ability

Tables 2–4 shows the FMA-UE, WMFT, and MBI scores for each group at different time points. Before treatment (baseline), there was no significant difference between FMA-UE, WMFT, and MBI scores in each group, which were comparable (all *p* > 0.05).

**TABLE 2:** Fugl-Meyer Assessment of the Upper Extremity (FMA-UE) score for all the groups at different time points ($\bar{\text{x}}$ ± S).

	Baseline	Post 1	Post 2	*F*	*p*
Group A (*n* = 15)	14.80 ± 4.38	22.80 ± 6.13*	38.80 ± 3.78*^#^	212.92	<0.001
Group B (*n* = 15)	15.47 ± 3.48	21.93 ± 4.92*	34.73 ± 5.48*^#^	137.96	<0.001
Group C (*n* = 15)	15.33 ± 3.99	20.73 ± 4.89*	33.53 ± 5.95*^#^	112.59	<0.001
Group D (*n* = 15)	14.93 ± 3.94	18.80 ± 4.69*	30.70 ± 5.65*^#^	71.09	<0.001
*F*	0.096	1.671	6.927		
*P*	0.962	0.184	<0.001		

FMA-UE, Fugl-Meyer Assessment Upper Extremity Scale.

*p < 0.01, compared with Baseline level.

^#^*p* < 0.01, compared with Post 1 level.

**TABLE 3:** Wolf Motor Function Test (WMFT) score for all the groups at different time points ($\bar{\text{x}}$ ± S).

	Baseline	Post 1	Post 2	*F*	*p*
Group A (*n* = 15)	12.53 ± 3.68	28.67 ± 5.84*	40.07 ± 6.41*^#^	315.23	<0.001
Group B (*n* = 15)	12.27 ± 3.90	25.00 ± 4.91*	35.93 ± 4.06*^#^	218.80	<0.001
Group C (*n* = 15)	11.60 ± 4.58	24.67 ± 4.82*	33.27 ± 4.09*^#^	199.92	<0.001
Group D (*n* = 15)	12.33 ± 4.67	20.40 ± 4.37*	31.87 ± 4.31*^#^	131.82	<0.001
*F*	0.138	6.82	8.41		
*p*	0.937	<0.001	<0.001		

WMFT, Wolf Motor Function Test; *p < 0.01, compared with the Baseline level.

^#^*p* < 0.01, compared with Post 1 level.

**TABLE 4:** Modified Barthel Index (MBI) score for all the groups at different time points ($\bar{\text{x}}$ ± S).

	Baseline	Post 1	Post 2	*F*	*p*
Group A (*n* = 15)	36.13 ± 9.47	58.40 ± 7.31*	87.07 ± 6.69*^#^	366.45	<0.001
Group B (*n* = 15)	35.33 ± 7.16	53.60 ± 7.73*	83.67 ± 7.42*^#^	322.61	<0.001
Group C (*n* = 15)	36.67 ± 7.11	52.27 ± 7.21*	81.67 ± 6.80*^#^	278.26	<0.001
Group D (*n* = 15)	35.87 ± 6.66	50.33 ± 8.09*	78.47 ± 7.76*^#^	249.26	<0.001
*F*	0.078	3.08	3.778		
*p*	0.971	0.035	0.015		

MBI, Modified Barthel Index.

*p < 0.01, compared with the Baseline level.

^#^*p* < 0.01, compared with Post 1 level.

Repeated measures of two-way ANOVA showed that FMA-UE score and WMFT score were significantly influenced by time (*F* = 513.69, *p* < 0.001 for FMA-UE; *F* = 875.86, *p* < 0.001 for WMFT), treatment (*F* = 3.13, *p* = 0.033 for FMA-UE; *F* = 5.09, *p* = 0.003 for WMFT) and by the time × treatment interaction (*F* = 5.19, *p* ≤ 0.001 for FMA-UE; *F* = 6.87, *p* ≤ 0.001 for WMFT). For MBI, there was a significant main effect for time (*F* = 1205.50, *p* < 0.001) along with a significant time × treatment interaction effect (*F* = 3.2, *p* = 0.006), but the main effects of the treatment interaction were not significant (*F* = 1.95, *p* = 0.132).

**FIGURE 3:**
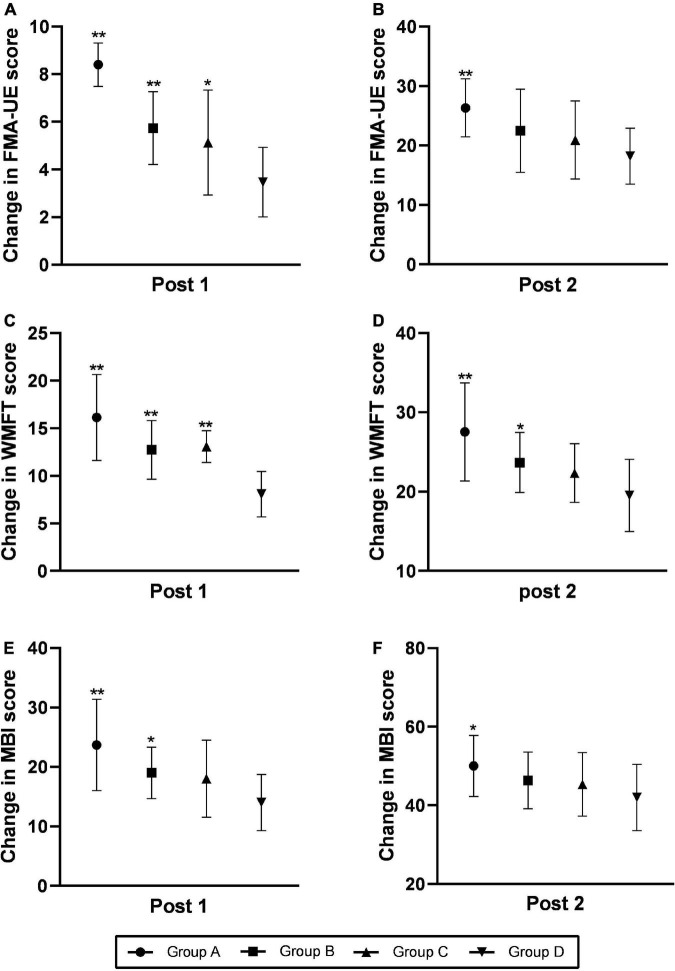
Comparison of changes in the FMA-UE **(A,B)**, WMFT **(C,D)**, and MBI **(E,F)** scores at the time of post-treatment (post 1) and the 3-month follow-up (post 2). Changes were calculated as follows: post-intervention value minus baseline value. The data were shown as the mean ± standard deviation values. Comparisons of value changes among the four groups were performed using ANOVA. *p < 0.05 and **p < 0.01 relative to group D. FMA-UE, Fugl-Meyer Assessment of the Upper Extremity; WMFT, Wolf Motor Function Test; MBI, Modified Barthel Index.

Figure 3 shows the Comparisons of changes in FMA-UE score, WMFT score and MBI score at the time of post-treatment (post 1) and 3-month follow up (post 2). The score of FMA-UE, WMFT, and MBI exhibited significantly improvement at post 1 and post 2. The improvement of group A was significantly better than the other three groups.

At post 1, there were no statistically significant differences in FMA-UE score between each group (*F* = 1.671, *p* = 0.184), but the differences in WMFT score (*F* = 6.82, *p* < 0.001) and MBI score (*F* = 3.08, *p* = 0.035)were significant. At post 2, the differences in FMA score (*F* = 6.927, *p* < 0.001), WMFT score (*F* = 8.41, *p* < 0.001), and MBI score (*F* = 3.771, *p* = 0.015) among those four groups were significant.

Subsequently, we use *post hoc* analysis to compare the four groups at three time points. *p*-values for the comparisons is showed in Table 5.

**TABLE 5:** *P*-values for the comparisons of FMA, WMFT, and MBI score in the four groups at different evaluation time points.

	FMA-UE score			WMFT score			MBI score		
	Baseline	Post 1	Post 2	Baseline	Post 1	Post 2	Baseline	Post 1	Post 2
Group A vs. Group B	0.647	0.649	0.040	0.864	0.050	0.018	0.776	0.089	0.200
Group A vs. Group C	0.714	0.280	0.008	0.548	0.033	<0.001	0.850	0.031	0.044
Group A vs. Group D	0.927	0.039	<0.001	0.897	<0.001	<0.001	0.925	0.005	0.022
Group B vs. Group C	0.927	0.529	0.537	0.668	0.856	0.242	0.636	0.632	0.449
Group B vs. Group D	0.714	0.104	0.019	0.966	0.015	0.019	0.850	0.243	0.052
Group C vs. Group D	0.783	0.312	0.078	0.637	0.023	0.227	0.776	0.448	0.228

FMA-UE. Fugl-Meyer Assessment Upper Extremity Scale; WMFT, Wolf Motor Function Test; MBI, Modified Barthel Index.

At post 1, group A manifested considerable improvement in FMA-UE score (*p* = 0.039), WMFT score (*p* < 0.001) and MBI score (*p* = 0.005) compared with group D. Group B and group C exhibited significant improvement in terms of WMFT score (*p* = 0.015 for group B, *p* = 0.023 for group C), but no significant changes were detected in the FMA-UE score (*p* = 0.104 for group B, *p* = 0.312 for group C) and MBI score (*p* = 0.243 for group B, *p* = 0.448 for group C) relative to the group D. There was no significant difference in FMA-UE score (*p* = 0.529), WMFT score (*p* = 0.856) and MBI (*p* = 0.632) scores between Group B and Group C.

At post 2, the group A exhibited significant improvements in terms of FMA scores (*p* < 0.001), WMFT score (*p* < 0.001) and MBI score (*p* = 0.022) relative to the group D. Compared with group D, group B manifested considerable improvements in terms of both the FMA-UE score (*p* = 0.019) and the WMFT score (*p* = 0.019), but no change were detected in MBI score (*p* = 0.052). There was no significant difference in FMA-UE score (*p* = 0.537), WMFT score (*p* = 0.242) and MBI score (*p* = 0.449) between group B and group C and between group C and group D.

### 3.2. Brunnstrom stage

Tables 6, 7 showed the Brunnstrom grading of upper limbs and hands of the four groups of patients at each evaluation time point. Before treatment, there was no significant difference in the motor function grading of the Brunnstrom stage among the four groups (*H* = 0.648, *p* = 0.885 for upper limb; *H* = 2.65, *p* = 0.499 for hand). At post 1 and post 2, the Brunnstrom motor function grades of upper limbs and hands in each group were improved, and the improvement of Group A was better than those of other groups, there is no statistical significant difference among all groups (*p* > 0.05).

**TABLE 6:** Brunnstrom Stage of each group–upper limb.

	Baseline [*n* (%)]			Post 1 [*n* (%)]		Post 2 [*n* (%)]		
	I	II	III	II	III	III	IV	V
Group A (*n* = 15)	5 (33.3)	9 (60.0)	1 (6.7)	2 (13.3)	13 (86.7)	1 (6.7)	9 (60.0)	5 (33.3)
Group B (*n* = 15)	5 (33.3)	10 (66.7)	0 (0)	8 (53.3)	7 (46.7)	3 (20.0)	9 (60.0)	3 (20.0)
Group C (*n* = 15)	4 (26.7)	11 (73.3)	0 (0)	4 (26.7)	11 (73.3)	2 (13.3)	8 (53.3)	5 (33.3)
Group D (*n* = 15)	6 (40.0)	9 (60.0)	0 (0)	8 (53.3)	7 (46.7)	6 (40.0)	8 (53.3)	1 (6.7)
*H*	0.648			7.622		7.39		
*P*	0.885			0.055		0.06		

**TABLE 7:** Brunnstrom Stage of each group–hand.

	Baseline [*n* (%)]		Post 1 [*n* (%)]			Post 2 [*n* (%)]			
	I	II	I	II	III	II	III	IV	V
Group A (*n* = 15)	8 (53.3)	7 (46.7)	1 (6.7)	9 (60.0)	5 (33.3)	1 (6.7)	8 (53.3)	5 (33.3)	1 (6.7)
Group B (*n* = 15)	11 (73.3)	4 (26.7)	3 (20.0)	11 (73.3)	1 (6.7)	2 (13.3)	8 (53.3)	5 (33.3)	0 (0)
Group C (*n* = 15)	10 (66.7)	5 (33.3)	2 (13.3)	9 (60.0)	4 (26.7)	1 (6.7)	7 (46.7)	6 (40.0)	1 (6.7)
Group D (*n* = 15)	12 (80.0)	3 (20.0)	5 (33.3)	9 (60.0)	1 (6.7)	4 (26.7)	9 (60.0)	2 (13.3)	0 (0)
*H*	2.65		7.15			6.01			
*P*	0.449		0.067			0.11			

### 3.3. Safety of the intervention

The four treatments protocol were well tolerated and all patients completed the treatment and follow-up. During the intervention, there was no significant change in vital signs. No patient experienced any adverse reactions, such as epilepsy, pain, or deterioration of the condition.

## 4. Discussion

The recovery of upper limb motor function has been a difficult issue for rehabilitation after stroke. Even though many stroke patients have undergone systematic rehabilitation treatment, the effect of upper limb function recovery is still poor. The application of rTMS improves the effect of upper limb functional rehabilitation after stroke (Hsu et al., 2012).

In this study, the FMA-UE score, WMFT score and MBI of the four groups were improved after treatment (post 1) and 3 months after treatment (post 2), which means the upper limb activity and the ADL of the patients in each group were improved. The increasement of group A, B, and C was higher than that of group D. At post 1, compared with group D, the increase rates of group A, B, and C were statistically significant (*p* < 0.05). At post 2, only the increasement of group A was significantly different from that of group D (*p* < 0.05). The results of this study showed that both HF-rTMS and cervical nerve root magnetic stimulation can effectively promote the recovery of upper limb function and improve ADL performance in stroke patients. The combination of the two was significantly more effective than either treatment alone or sham, and the therapeutic advantages can last for 3 months.

It is well known that cortical spinal tract (CST) plays a critical role in motor recovery after stroke, specifically fine motor control of upper limb and finger (Sterr et al., 2014; Rondina et al., 2017). Stroke disrupts neural circuit connectivity, which results in long-term neurological disability. RTMS can mediate the recovery of motor function after stroke by inducing cortical reorganization and regulating the excitability of corticospinal tract through neural plasticity (Auriat et al., 2015). RTMS modulates neuroplasticity not only locally below the magnetic coil but also in remote cortical and subcortical regions through functional connectivity of motor network (Cheng et al., 2014).

In this study, we used high-frequency (10 H z) rTMS because it has been reported that HF-rTMS have a more effective impact when compared to LF-rTMS (Sasaki et al., 2013; Caglayan et al., 2019). Kim et al. (2006) study demonstrated HF-rTMS of the affected motor cortex can produce increase in corticospinal excitability, facilitate practice-dependent plasticity and improve the motor learning performance in chronic stroke victims. Hong et al. (2020) shown that 10 Hz rTMS on the ipsilateral M1 can reduce infarct volume and promote functional recovery by inhibiting neurotoxic effects and reversing neuroprotective effects on astrocytes after ischemia/reperfusion injury in rats. Another study (Hong et al., 2022) showed that HF-rTMS could inhibit M1 polarization of microglia after cerebral ischemia-reperfusion injury and alleviate ischemic stroke injury via regulating leT-7B-5P/HMGA2/NF-κB signaling pathway. A systematic review (Vabalaite et al., 2021) showed that HF-rTMS increased impaired upper limb motor function better than sham stimulation in stroke patients. Moslemi Haghighi et al. (2021) also showed that HF-rTMS could effectively improve upper limb function for hemiplegia patients in the subacute stage of stroke. In addition, rTMS can protect nerves, supply nerve nutrition, promote nerve repair and regeneration, and reduce infarct area (van Lieshout et al., 2020).

Peripheral magnetic stimulation (Struppler et al., 2007) induces proprioceptive input of the central nervous system by directly stimulating peripheral sensorimotor afferent nerves or indirectly stimulating mechanical receptors by stimulating the muscle to produce rhythmic contraction and vibration. Gallasch et al. (2015) found that rPMS can induce the activation of sensorimotor networks and the changes of corticomotor excitability. Litvak’s experiment (Litvak et al., 2007) proved that magnetic stimulation of muscles or peripheral nerves can help the upper limb motor function recovery of stroke patients by promoting the plasticity change of M1 region and providing sensory input. The study of Chen et al. (2020) proved that PMS can improve the Fugl-Meyer score and Barthel index of upper limbs in stroke patients, and the efficacy is superior to LF-RTMS. Struppler et al. (2007) study on eight patients with mild paralysis after focal cerebral ischemia found that after repeated PMS, the recovery of hand function and the decrease of spasticity were related to the significant increase of neural activation within the superior posterior parietal lobe and the premotor cortex areas. Ke et al. (2022) randomized controlled trial showed that high-frequency (20 Hz) repetitive peripheral magnetic can significantly improve motor function and proximal muscle strength of the upper and lower limbs of patients with intracerebral hemorrhage. An animal experiment (Zheng et al., 2022) found that the nerve root magnetic stimulation enhanced nerve conduction in the injured spinal cord and promoted the recovery of synaptic ultrastructure in the sensorimotor cortex. These results indicate that PMS can regulate cerebral cortex function, improve brain plasticity, and have positive significance in improving muscle tone, limb function, muscle strength, and daily living activities after stroke. In addition, compared with rTMS, PMS was generally safe and tolerable for patients with stroke (Beaulieu and Schneider, 2015).

The present scheme is similar to paired associative stimulus. Paired associative stimulation (PAS) is a stimulation method pairing TMS with peripheral electrical stimulus (Stefan et al., 2002), which first reported by Stefan. PAS has been shown to elicit neural plasticity, enhance nerve conduction and promote function recovery after stroke, which has been widely used in stroke patients as an recovery treatment. In the present study, we modified the PAS protocol, and proposed the paired associative magnetic stimulation (PAMS) (Sun et al., 2022) protocol, which combines PMS and HF-rTMS of the ipsilesional sensorimotor cortex. Compared with peripheral electrical stimulation (PES), PMS has the following advantages. PMS generates a greater proprioceptive inflow through recruitment of muscle and joint afferents (Beaulieu and Schneider, 2015), provide a greater range of depth and less pain, and without removing clothes (Rossini et al., 2015), so it is easier to be implemented in clinical settings. Another main difference is that PAS activates the sensory and motor system simultaneously and induces spike timing dependent plasticity (Brzosko et al., 2019), however, our protocol applying the cortical and peripheral stimulation consecutively.

The protocol in present study is a novel magnetic stimulation protocol, targeting the motor cortex and the spinal nerve roots. On one hand, rTMS activates the cerebral cortex of the ipsilateral side, promotes the downward projection of the corticospinal tract, and may improves the plasticity of the nerve. On the other hand, peripheral nerve stimulation can enhance peripheral sensory and motor input and feedback, and promote the ascending pathway from nerve root to the cortex. Central intervention and peripheral intervention are organically combined to form closed-loop information feedback (Liu et al., 2022), to enhance the plasticity of the brain and the remodeling of neural pathways, promote the recovery of upper limb motor function and improve rehabilitation efficiency. An animal experiment (Gao et al., 2020) showed that PAMS activates the ipsilateral sensorimotor and sensory cortex, and that it upregulates the expression of brain plasticity-related proteins to ultimately change behavior. Kumru et al. (2017) about eleven healthy subjects found that PAMS can increase corticospinal excitability and reduce intracortical inhibition, but the effects were not present when the PMS and LF-rTMS were applied separately. These might be relevant for motor rehabilitation. In addition, the cervical nerve root magnetic stimulation can stimulate the spinothalamic tract up and the peripheral nerve down, to achieve bidirectional regulation of nerve effect. In conclusion, this treatment may be a valuable treatment for stroke patients. However, due to the multiple parameters of TMS and PMS, the combination of different parameters can produce different therapeutic effects, so how formulating the optimal treatment prescription to achieve the best therapeutic effect for patients is worthy of further study. At the same time, the sample size of this study was small and the observation time was short, so the mechanism of action of this scheme could not be further studied through other auxiliary examinations. In the future, we will further expand the sample size and further study the effect of this treatment plan on neurological function recovery combined with functional magnetic resonance and electrophysiological examination, to provide a reference for clinical application.

## 5. Conclusion

Both 10 Hz rTMS to the ipsilateral M1 and cervical nerve root magnetic stimulation to the hemiplegic side can effectively promote the recovery of upper limb function in patients with stroke. The protocol combining the two is more beneficial for motor improvement and patients can easily tolerate it. This protocol is worthy of clinical application.

## Data availability statement

The datasets presented in this study can be found in online repositories. The names of the repository/repositories and accession number(s) can be found in the article/supplementary material.

## Ethics statement

The studies involving human participants were reviewed and approved by Ethics Committee of Tangdu Hospital of Air Force Military Medical University. The patients/participants provided their written informed consent to participate in this study. The animal study was reviewed and approved by Ethics Committee of Tangdu Hospital of Air Force Military Medical University.

## Author contributions

RL and XW contributed to the conception and design of the study. QW, CL, JZ, and HJ carried out the experiments. BC and SW performed the statistical analysis. XW and RW wrote the manuscript. All authors read and approved the final manuscript.

## References

[B1] Auriat A. M. Neva J. L. Peters S. Ferris J. K. Boyd L. A. (2015). A review of transcranial magnetic stimulation and multimodal neuroimaging to characterize post-stroke neuroplasticity. *Front. Neurol.* 6:226. 10.3389/fneur.2015.00226 26579069 PMC4625082

[B2] Beaulieu L. D. Schneider C. (2015). Repetitive peripheral magnetic stimulation to reduce pain or improve sensorimotor impairments: A literature review on parameters of application and afferents recruitment. *Neurophysiol. Clin.* 45 223–237. 26363684 10.1016/j.neucli.2015.08.002

[B3] Bornheim S. Croisier J. L. Maquet P. Kaux J. F. (2020). Transcranial direct current stimulation associated with physical-therapy in acute stroke patients - A randomized, triple blind, sham-controlled study. *Brain Stimul.* 13 329–336. 10.1016/j.brs.2019.10.019 31735645

[B4] Brzosko Z. Mierau S. B. Paulsen O. (2019). Neuromodulation of spike-timing-dependent plasticity: Past, present, and future. *Neuron* 103 563–581. 10.1016/j.neuron.2019.05.041 31437453

[B5] Caglayan A. B. Beker M. C. Caglayan B. Yalcin E. Caglayan A. Yulug B. (2019). Acute and post-acute neuromodulation induces stroke recovery by promoting survival signaling, neurogenesis, and pyramidal tract plasticity. *Front. Cell. Neurosci.* 13:144. 10.3389/fncel.2019.00144 31031599 PMC6474396

[B6] Chen X. Liu X. Cui Y. Xu G. Liu L. Zhang X. (2020). Efficacy of functional magnetic stimulation in improving upper extremity function after stroke: A randomized, single-blind, controlled study. *J. Int. Med. Res.* 48:30006052092 7881. 10.1177/0300060520927881 PMC7273768 32495667

[B7] Cheng M. Y. Wang E. H. Woodson W. J. Wang S. Sun G. Lee A. G. (2014). Optogenetic neuronal stimulation promotes functional recovery after stroke. *Proc. Natl. Acad. Sci. U.S.A.* 111 12913–12918. 25136109 10.1073/pnas.1404109111 PMC4156770

[B8] Gallasch E. Christova M. Kunz A. Rafolt D. Golaszewski S. (2015). Modulation of sensorimotor cortex by repetitive peripheral magnetic stimulation. *Front. Hum. Neurosci.* 9:407. 10.3389/fnhum.2015.00407 26236220 PMC4500907

[B9] Gao B. Y. Sun C. C. Xia G. H. Zhou S. T. Zhang Y. Mao Y. R. (2020). Paired associated magnetic stimulation promotes neural repair in the rat middle cerebral artery occlusion model of stroke. *Neural Regen. Res.* 15 2047–2056. 10.4103/1673-5374.282266 32394960 PMC7716041

[B10] Hernandez E. D. Galeano C. P. Barbosa N. E. Forero S. M. Nordin A. Sunnerhagen K. S. (2019). Intra- and inter-rater reliability of fugl-meyer assessment of upper extremity in stroke. *J. Rehabil. Med.* 51 652–659. 31448807 10.2340/16501977-2590

[B11] Hong Y. Liu Q. Peng M. Bai M. Li J. Sun R. (2020). High-frequency repetitive transcranial magnetic stimulation improves functional recovery by inhibiting neurotoxic polarization of astrocytes in ischemic rats. *J. Neuroinflammation* 17:150. 10.1186/s12974-020-01747-y 32375835 PMC7203826

[B12] Hong Y. Lyu J. Zhu L. Wang X. Peng M. Chen X. (2022). High-frequency repetitive transcranial magnetic stimulation (rTMS) protects against ischemic stroke by inhibiting M1 microglia polarization through let-7b-5p/HMGA2/NF-kappaB signaling pathway. *BMC Neurosci.* 23:49. 10.1186/s12868-022-00735-7 35927640 PMC9351069

[B13] Hsu W. Y. Cheng C. H. Liao K. K. Lee I. H. Lin Y. Y. (2012). Effects of repetitive transcranial magnetic stimulation on motor functions in patients with stroke: A meta-analysis. *Stroke* 43 1849–1857. 10.1161/STROKEAHA.111.649756 22713491

[B14] Ikbali Afsar S. Mirzayev I. Umit Yemisci O. Cosar Saracgil S. N. (2018). Virtual reality in upper extremity rehabilitation of stroke patients: A randomized controlled trial. *J. Stroke Cerebrovasc. Dis.* 27 3473–3478. 10.1016/j.jstrokecerebrovasdis.2018.08.007 30193810

[B15] Jiang Y. F. Zhang D. Zhang J. Hai H. Zhao Y. Y. Ma Y. W. (2022). A randomized controlled trial of repetitive peripheral magnetic stimulation applied in early subacute stroke: Effects on severe upper-limb impairment. *Clin. Rehabil.* 36 693–702. 10.1177/02692155211072189 34985366

[B16] Ke J. Wei J. Zheng B. Tan T. Zhou W. Zou X. (2022). Effect of high-frequency repetitive peripheral magnetic stimulation on motor performance in intracerebral haemorrhage: A clinical trial. *J. Stroke Cerebrovasc. Dis.* 31:106446. 10.1016/j.jstrokecerebrovasdis.2022.106446 35512466

[B17] Kim Y. H. You S. H. Ko M. H. Park J. W. Lee K. H. Jang S. H. (2006). Repetitive transcranial magnetic stimulation-induced corticomotor excitability and associated motor skill acquisition in chronic stroke. *Stroke* 37 1471–1476. 10.1161/01.STR.0000221233.55497.51 16675743

[B18] Klomjai W. Katz R. Lackmy-Vallee A. (2015). Basic principles of transcranial magnetic stimulation (TMS) and repetitive TMS (rTMS). *Ann. Phys. Rehabil. Med.* 58 208–213. 26319963 10.1016/j.rehab.2015.05.005

[B19] Kumru H. Albu S. Rothwell J. Leon D. Flores C. Opisso E. (2017). Modulation of motor cortex excitability by paired peripheral and transcranial magnetic stimulation. *Clin. Neurophysiol.* 128 2043–2047. 10.1016/j.clinph.2017.06.041 28858700

[B20] Lefaucheur J. P. Andre-Obadia N. Antal A. Ayache S. S. Baeken C. Benninger D. H. (2014). Evidence-based guidelines on the therapeutic use of repetitive transcranial magnetic stimulation (rTMS). *Clin. Neurophysiol.* 125 2150–2206. 25034472 10.1016/j.clinph.2014.05.021

[B21] Li J. Meng X. M. Li R. Y. Zhang R. Zhang Z. Du Y. F. (2016). Effects of different frequencies of repetitive transcranial magnetic stimulation on the recovery of upper limb motor dysfunction in patients with subacute cerebral infarction. *Neural Regen. Res.* 11 1584–1590. 10.4103/1673-5374.193236 27904488 PMC5116836

[B22] Litvak V. Zeller D. Oostenveld R. Maris E. Cohen A. Schramm A. (2007). LTP-like changes induced by paired associative stimulation of the primary somatosensory cortex in humans: Source analysis and associated changes in behaviour. *Eur. J. Neurosci.* 25 2862–2874. 10.1111/j.1460-9568.2007.05531.x 17561848

[B23] Liu J. Tan G. Wang J. Wei Y. Sheng Y. Chang H. (2022). Closed-loop construction and analysis of cortico-muscular-cortical functional network after stroke. *IEEE Trans. Med. Imaging* 41 1575–1586. 10.1109/TMI.2022.3143133 35030075

[B24] Matsumoto H. Hanajima R. Terao Y. Ugawa Y. (2013). Magnetic-motor-root stimulation: Review. *Clin. Neurophysiol.* 124 1055–1067. 23485367 10.1016/j.clinph.2012.12.049

[B25] Micera S. Caleo M. Chisari C. Hummel F. C. Pedrocchi A. (2020). Advanced neurotechnologies for the restoration of motor function. *Neuron* 105 604–620. 32078796 10.1016/j.neuron.2020.01.039

[B26] Moslemi Haghighi F. Kordi Yoosefinejad A. Razeghi M. Shariat A. Bagheri Z. Rezaei K. (2021). The effect of high-frequency repetitive transcranial magnetic stimulation on functional indices of affected upper limb in patients with subacute stroke. *J. Biomed. Phys. Eng.* 11 175–184. 10.31661/jbpe.v0i0.879 33937125 PMC8064128

[B27] Naghavi M. Abajobir A. A. Abbafati C. Abbas K. M. Abd-Allah F. Abera S. F. (2017). Global, regional, and national age-sex specific mortality for 264 causes of death, 1980–2016: A systematic analysis for the Global Burden of Disease Study 2016. *Lancet* 390 1151–1210. 10.1016/S0140-6736(17)32152-9 28919116 PMC5605883

[B28] Ohura T. Hase K. Nakajima Y. Nakayama T. (2017). Validity and reliability of a performance evaluation tool based on the modified Barthel Index for stroke patients. *BMC Med. Res. Methodol.* 17:131. 10.1186/s12874-017-0409-2 28841846 PMC6389202

[B29] Rondina J. M. Park C. H. Ward N. S. (2017). Brain regions important for recovery after severe post-stroke upper limb paresis. *J. Neurol. Neurosurg. Psychiatry* 88 737–743. 10.1136/jnnp-2016-315030 28642286 PMC5561379

[B30] Rossi S. Antal A. Bestmann S. Bikson M. Brewer C. Brockmoller J. (2021). Safety and recommendations for TMS use in healthy subjects and patient populations, with updates on training, ethical and regulatory issues: Expert Guidelines. *Clin. Neurophysiol.* 132 269–306. 10.1016/j.clinph.2020.10.003 33243615 PMC9094636

[B31] Rossini P. M. Burke D. Chen R. Cohen L. G. Daskalakis Z. Di Iorio R. (2015). Non-invasive electrical and magnetic stimulation of the brain, spinal cord, roots and peripheral nerves: Basic principles and procedures for routine clinical and research application. An updated report from an I.F.C.N. committee. *Clin. Neurophysiol.* 126 1071–1107. 10.1016/j.clinph.2015.02.001 25797650 PMC6350257

[B32] Sasaki N. Mizutani S. Kakuda W. Abo M. (2013). Comparison of the effects of high- and low-frequency repetitive transcranial magnetic stimulation on upper limb hemiparesis in the early phase of stroke. *J. Stroke Cerebrovasc. Dis.* 22 413–418. 10.1016/j.jstrokecerebrovasdis.2011.10.004 22177936

[B33] Stefan K. Kunesch E. Benecke R. Cohen L. G. Classen J. (2002). Mechanisms of enhancement of human motor cortex excitability induced by interventional paired associative stimulation. *J. Physiol.* 543 699–708. 12205201 10.1113/jphysiol.2002.023317 PMC2290505

[B34] Sterr A. Dean P. J. Szameitat A. J. Conforto A. B. Shen S. (2014). Corticospinal tract integrity and lesion volume play different roles in chronic hemiparesis and its improvement through motor practice. *Neurorehabil. Neural Repair* 28 335–343. 10.1177/1545968313510972 24334657

[B35] Struppler A. Binkofski F. Angerer B. Bernhardt M. Spiegel S. Drzezga A. (2007). A fronto-parietal network is mediating improvement of motor function related to repetitive peripheral magnetic stimulation: A PET-H2O15 study. *Neuroimage* 36(Suppl. 2), T174–T186. 10.1016/j.neuroimage.2007.03.033 17499165

[B36] Sun T. T. Zhu G. Y. Zheng Y. Mao Y. R. Hu Q. L. Song G. M. (2022). Effects of paired associative magnetic stimulation between nerve root and cortex on motor function of lower limbs after spinal cord injury: Study protocol for a randomized controlled trial. *Neural Regen. Res.* 17 2459–2464. 10.4103/1673-5374.339012 35535897 PMC9120678

[B37] Vabalaite B. Petruseviciene L. Savickas R. Kubilius R. Ignatavicius P. Lendraitiene E. (2021). Effects of high-frequency (HF) repetitive transcranial magnetic stimulation (rTMS) on upper extremity motor function in stroke patients: A systematic review. *Medicina* 57:1215. 10.3390/medicina57111215 PMC8617907 34833433

[B38] van Lieshout E. C. Jacobs L. D. Pelsma M. Dijkhuizen R. M. Visser-Meily J. M. (2020). Exploring the experiences of stroke patients treated with transcranial magnetic stimulation for upper limb recovery: A qualitative study. *BMC Neurol.* 20:365. 10.1186/s12883-020-01936-5 33023487 PMC7541313

[B39] Winstein C. J. Stein J. Arena R. Bates B. Cherney L. R. Cramer S. C. (2016). Guidelines for adult stroke rehabilitation and recovery: A guideline for healthcare professionals from the American heart association/American stroke association. *Stroke* 47 e98–e169. 27145936 10.1161/STR.0000000000000098

[B40] Yang Y. Pan H. Pan W. Liu Y. Song X. Niu C. M. (2021). Repetitive transcranial magnetic stimulation on the affected hemisphere enhances hand functional recovery in subacute adult stroke patients: A randomized trial. *Front. Aging Neurosci.* 13:636184. 10.3389/fnagi.2021.636184 34093164 PMC8171119

[B41] Zhang T. Zhao J. Li X. Bai Y. Wang B. Qu Y. (2020). Chinese stroke association guidelines for clinical management of cerebrovascular disorders: Executive summary and 2019 update of clinical management of stroke rehabilitation. *Stroke Vasc. Neurol.* 5 250–259. 32595138 10.1136/svn-2019-000321 PMC7548515

[B42] Zheng Y. Zhao D. Xue D. D. Mao Y. R. Cao L. Y. Zhang Y. (2022). Nerve root magnetic stimulation improves locomotor function following spinal cord injury with electrophysiological improvements and cortical synaptic reconstruction. *Neural Regen. Res.* 17 2036–2042. 10.4103/1673-5374.335161 35142694 PMC8848603

